# Divalent Metal Transporter 1 Knock-Down Modulates IL-1β Mediated Pancreatic Beta-Cell Pro-Apoptotic Signaling Pathways through the Autophagic Machinery

**DOI:** 10.3390/ijms22158013

**Published:** 2021-07-27

**Authors:** Taewook Kang, Honggang Huang, Thomas Mandrup-Poulsen, Martin R. Larsen

**Affiliations:** 1 Protein Research Group, Department of Biochemistry and Molecular Biology, University of Southern Denmark, 5230 Odense M, Denmark; taewookk@bmb.sdu.dk (T.K.); hongganghuang@hotmail.com (H.H.); 2 The Danish Diabetes Academy, 5230 Odense M, Denmark; 3 Immuno-Endocrinology Laboratory, Department of Biomedical Sciences, University of Copenhagen, 2200 Copenhagen N, Denmark; tmpo@sund.ku.dk

**Keywords:** diabetes, iron metabolism, autophagy, ROS, cell cycle arrest, anti-apoptosis

## Abstract

Pro-inflammatory cytokines promote cellular iron-import through enhanced divalent metal transporter-1 (*DMT1*) expression in pancreatic β-cells, consequently cell death. Inhibition of β-cell iron-import by *DMT1* silencing protects against apoptosis in animal models of diabetes. However, how alterations of signaling networks contribute to the protective action of *DMT1* knock-down is unknown. Here, we performed phosphoproteomics using our sequential enrichment strategy of mRNA, protein, and phosphopeptides, which enabled us to explore the concurrent molecular events in the same set of wildtype and *DMT1*-silenced β-cells during IL-1β exposure. Our findings reveal new phosphosites in the IL-1β-induced proteins that are clearly reverted by *DMT1* silencing towards their steady-state levels. We validated the levels of five novel phosphosites of the potential protective proteins using parallel reaction monitoring. We also confirmed the inactivation of autophagic flux that may be relevant for cell survival induced by *DMT1* silencing during IL-1β exposure. Additionally, the potential protective proteins induced by *DMT1* silencing were related to insulin secretion that may lead to improving β-cell functions upon exposure to IL-1β. This global profiling has shed light on the signal transduction pathways driving the protection against inflammation-induced cell death in β-cells after *DMT1* silencing.

## 1. Introduction

In pancreatic β-cells, iron plays a critical role as a cofactor for normal development, free fatty acid and glucose oxidation, electron transport and insulin secretion [1,2,3]. Iron overload as a consequence of, e.g., hereditary hemochromatosis or the transfusion syndrome excessively generates reactive oxygen species (ROS) and subsequent oxidative stress that are harmful to the cells, triggering functional defects or demise [1,2,3,4], leading to inadequate insulin secretion to meet metabolic demands and diabetes in these patients [5,6,7].

Islet inflammation including aberrant activation of islet-resident macrophages contributes to the pathogenesis of both common forms of diabetes, Type 1 and Type 2 diabetes (T1D and T2D) [8,9,10]. Activated islet inflammatory cells produce pro-inflammatory cytokines (e.g., interleukin-1 beta (IL-1β), tumor necrosis factor (TNF), and interferon (IFN)) now recognized as major mediators of β-cell failure and apoptosis, in part by ROS and reactive nitrogen species (RNS) [10,11,12,13,14].

IL-1β in synergy with other pro-inflammatory cytokines reprograms β-cell gene expression, one of which is divalent metal transporter 1 (*DMT1*; also known as *SLC11A2* or *NRAMP2*) in a nuclear factor kappa beta (NF-κB) dependent fashion, causing enhanced β-cell iron uptake and increased ROS production, resulting in β-cell failure and subsequent apoptosis-mediated cell death [1].

Interestingly, iron chelation or *DMT1* genetic silencing by small interfering RNAs (siRNAs) in vitro or inducible β-cell-specific knock-out (KO) in mice attenuate IL-1β-mediated ROS formation and protect β-cells against apoptosis upon exposure to pro-inflammatory cytokines or glucolipotoxic conditions [1]. Of note, *DMT1*-deficient β-cells have slightly impaired glucose-stimulated insulin secretion [1] due to the decrease in ROS signaling from mitochondrial glucose-oxidation that fine-tunes the stimulus-secretion coupling, but this is compensated for in vivo, and *DMT1* KO mice do not exhibit a phenotype under unchallenged condition [2,15]. However, how reprogramming of global signaling networks downstream of cytokine-regulated DMT1 activity contributes to inflammatory β-cell death is unknown. Such a global profiling may have translational relevance as it may guide the discovery of β-cell-specific diagnostic and therapeutic targets for diabetes.

To investigate the downstream molecular events responsible for the protective effects observed upon iron chelation during pro-inflammatory cytokine exposure of ß-cells, we silenced *DMT1* in the pancreatic ß-cell line INS-1E prior to exposure to IL-1ß. Then we applied a sequential sample preparation and enrichment strategy of mRNA, protein, and phosphopeptides to characterize the DTM1 knock-down, the proteome and the phosphoproteome from the same set of samples. For the quantitative proteomics and phosphoproteomics analysis, we used a combination of our TiO_2_-SIMAC method (enrichment of mono- and multi-phosphorylated peptides), tandem mass tags (TMT) labeling, and a nano-liquid chromatography-tandem mass spectrometry (nLC-MS/MS) approach [16,17,18,19]. Overall, in addition to identifying canonical pathways for IL-1 receptor signaling, NF-κB activation pathway, and inflammatory response, we identified two novel pathways containing apoptotic and anti-apoptotic proteins that are involved in cell survival. We also uncovered new phosphosites of proteins associated with the regulation of apoptosis, ROS production, cell cycle arrest, and autophagy that were restored to normal levels by *DMT1* silencing in IL-1β exposed cells. We confirmed the inactivation of autophagic flux that promotes cell survival mediated by *DMT1* silencing during IL-1β exposure in INS-1E. Furthermore, we conducted parallel reaction monitoring (PRM) using isotopically labeled synthetic phosphopeptides to validate the regulation of a subset of phosphopeptides and found a high correlation with the TMT analysis, verifying the quality of the large-scale analysis. Collectively, our study provides new insights at the molecular level into the potential protective mechanisms against ROS/RNS-induced autophagy by *DMT1* silencing for improving β-cell function and viability of high relevance to enhance our understanding of β-cell death and survival.

## 2. Results and Discussion

### 2.1. Overview of High-Throughput Analysis after DMT1 Knock-Down in Response to IL-1ß in INS-1E Cells

**Figure 1:**
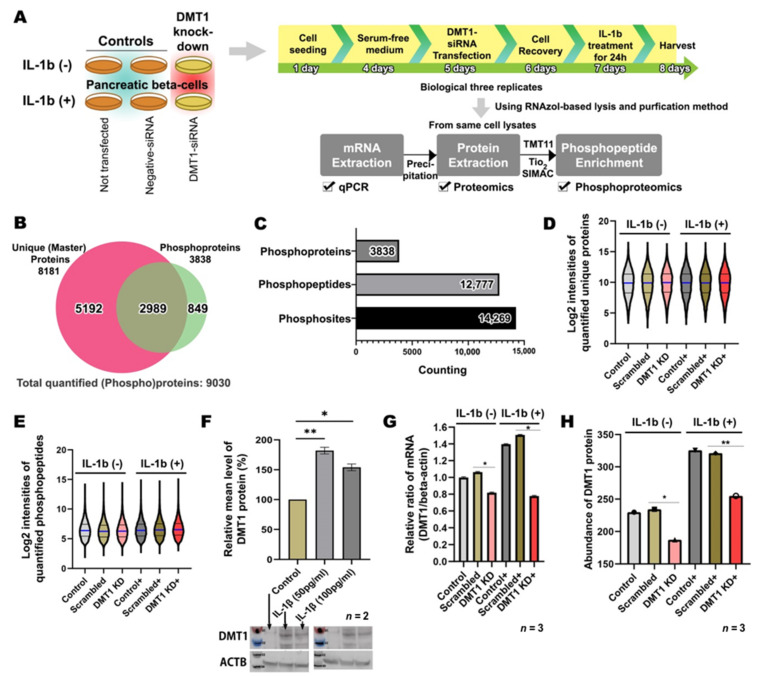
In-depth proteomics and phosphoproteomics profiling of the potential protective effect of iron depletion on inflammation-mediated β-cell dysfunction (**A**) Schematic of experimental designs for integrated proteomics and phosphoproteomics using a sequential enrichment strategy of mRNA, protein, and phosphopeptides occurring from the same sample. (**B**) Venn diagram representing the overlap between unique (master) proteins and phosphoproteins in β-cells. Approximately 76% of phosphoproteins are overlapped between proteome and phosphoproteome. Unique proteins are not identified in another protein accession number. Unique proteins and phosphopeptides are quantified for further analysis. (**C**) Histogram showing a total of 12,777 phosphopeptides carrying 14,269 high-confident phosphosites (75% < phosphosite localization probability) on 3838 phosphoproteins. (**D**) Violin plot showing the overall distribution of the normalized abundance of quantified unique proteins across six experimental groups. (Blue line: median) (**E**) Violin plot showing the overall distribution of the normalized abundance of quantified phosphopeptides across six experimental groups. (Blue line: median) (**F**) Western blotting finds significantly increased protein production of DMT1 after exposure to IL-1β (50 and 100 pg/mL). *n* = 2 biological replicates (**G**) qPCR verify markedly enhanced mRNA expression of *DMT1* after IL-1β treatment (50 pg/mL). *n* = 3 biological replicates (**H**) Proteomics shows the correlation with qPCR results, which DMT1 protein production is increased in the IL-1β treated β-cell and decreased in *DMT1* knock-down β-cells with IL-1β concentrations. *n* = 3 biological replicates. * *p* < 0.05; ** *p* < 0.01.

To characterize the signaling mechanism of the protective effects of *DMT1* silencing upon β-cell cytokine exposure, we applied a high-throughput quantification approach by nLC-MS based proteomics and phosphoproteomics in conjunction with TMT labeling, as well as bioinformatics and validation using PRM (Figure 1A) [16,17,18,19]. For consistent results at protein and mRNA levels, we modified our TiSH protocol [16,18] resulting in a sequential mRNA, protein, and phosphopeptide enrichment strategy, which enabled us to simultaneously investigate molecular events derived from the same sample. As expected, our method led to high correlation of *DMT1* levels quantified from qPCR and proteome datasets. The mRNA and protein levels of *DMT1* had similar patterns of expression levels, confirmed by a Pearson’s correlation in three biological replicates (r = 0.80, log_2_ ratio-ratio).

In agreement with our strategy, we analyzed the proteome and phosphoproteome of six experimental groups harvested from un-transfected control cells (CON), scrambled siRNA transfected control cells (SC), and *DMT1* siRNA transfected β-cells (*DMT1*-KD) that were cultured for 24 h either with or without exposure to 50 pg/mL of IL-1β (Figure 1A). Our integrated methods enabled us to identify a total of 9030 proteins (including phosphoproteins) in β-cells, together with exploring a total of 12,777 unique phosphopeptides carrying 14,269 high-confident phosphosites (acceptable phosphosite localization probability cutoff of 75% [20]) on 3838 unique phosphoproteins and to explore their regulation during IL-1β exposure in control versus *DMT1*-silenced β-cells (Figure 1B,C). Of these, 87% of the phosphosites were distinctively monitored with highly reliable localization of phosphosites (99% < PhosphoRS site probability) at a single amino acid (S,T,Y) on the unique peptide backbone. In total, 76% of phosphoproteins were overlapping between the proteome and the phosphoproteome (Figure 1B), which enabled normalization of the phosphoproteome based on the protein expression level. Proteome quantitative measurements were appropriately normalized by standard deviation factors ranging between 0.9 and 1.1 for each sample, which were estimated to be technically reproducible and accurate. For normalization of the phosphosite abundance to the protein abundance, we used standard deviation factors on each group calculated by the total of non-phosphorylated proteins abundance. The distribution of the normalized abundance of proteins or phosphopeptides was evaluated across the six experimental groups for relative quantification, and they were appropriately equivalent to each sample (Figure 1D,E). These data show that our method can be used to reliably quantify global occurring changes at mRNA, protein, and posttranslational modifications levels in the same sample.

We evaluated the effects of two conditions (INS-1E in the presence or absence of IL-1β) using Western blotting and qPCR. In response to IL-1β exposure (50 and 100 pg/mL), the insulin-producing cells exhibited significantly increased *DMT1* expression at the mRNA and protein levels when compared to control, confirming earlier findings [15] (Figure 1F,G). Furthermore, the siRNA-induced silencing of *DMT1* was verified at the mRNA level in the *DMT1*-KD and IL-1β-exposed *DMT1*-silenced cells (*DMT1*-KD-IL) (Figure 1G). Likewise, DMT1 protein production was significantly increased in the IL-1β-treated β-cells (CON-IL: control with IL-1β and SC-IL: scrambled cells with IL-1β; mRNA level: 1.42 fold, *p*-value = 0.0455; protein level: 1.31 fold, *p*-value = 0.0233 in SC-IL versus SC), and the reduced degree of *DMT1* expression in the *DMT1*-KD-IL (mRNA level: 0.52 fold, *p*-value = 0.0259; protein level: 0.72 fold, *p*-value = 0.0056 in *DMT1*-KD-IL versus SC-IL; Figure 1H), as previously reported [1].

**Figure 2:**
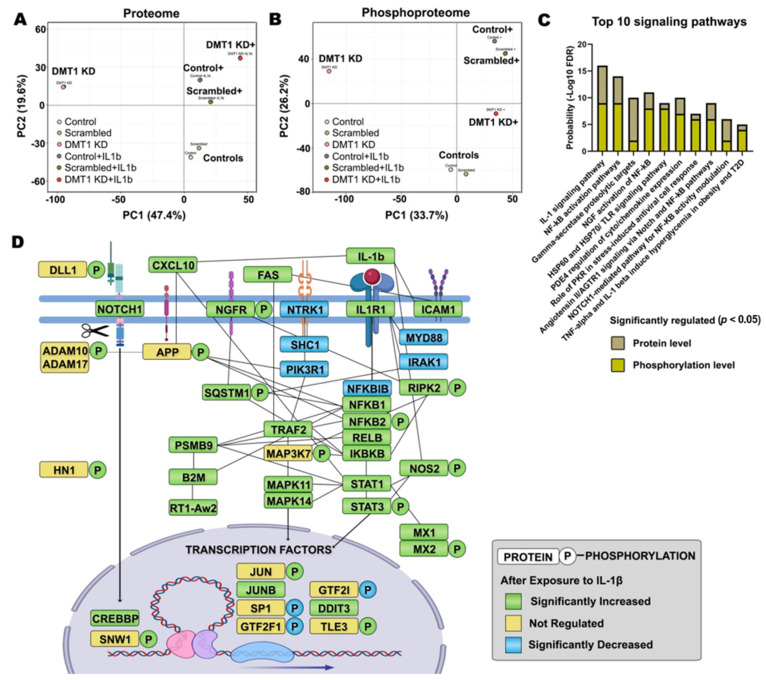
The pro-inflammatory effect in IL-1β exposed pancreatic β-cells. (**A**) PCA plots of the averaged proteome profiles of Control (−) (light grey), Scrambled (−) (light brown), *DMT1* KD (−) (pink), Control + IL-1β (dark grey), Scrambled + IL-1β (dark brown), *DMT1* KD + IL1b (red) cells. (**B**) PCA plots of the averaged phosphoproteome profiles of Control (−) (light grey), Scrambled (−) (light brown), *DMT1* KD (−) (pink), Control + IL-1β (dark grey), Scrambled + IL-1β (dark brown), *DMT1* KD + IL-1β (red) cells. (**C**) Significantly enriched top 10 signaling pathways (FDR < 0.05) are classified in regulated proteins and phosphopeptides in the comparison of SC and SC-IL. (beige bar: probability from the proteome, gold bar: probability from the phosphoproteome) (**D**) protein-protein interaction network across IL-1β-regulated proteins and protein phosphorylation. P, phosphosite. (green: significantly increased, blue: significantly decreased, yellow: not significantly regulated).

We next assessed the overall characteristics of the global molecular changes in the six experimental groups. For this, we first conducted principal component analysis (PCA) of both our proteome and phosphoproteome datasets. In the multiplexing quantification, the six groups for the averaged relative percentages of each protein/phosphopeptide abundance were transformed into pairs of variables (PC1 and PC2) and visualized in a plot chart (Figure 2A,B). The CON or CON-IL groups were clustered closely together with the SC or SC-IL groups, respectively. Nonetheless, there is still a small difference between the controls, which explains why the SC or SC-IL groups were found appropriate as control groups for the comparative analysis with *DMT1*-KD and *DMT1*-KD-IL. However, to remove the common interfering effect of siRNA transfection, we only accepted the proteins (89% of total proteins) and phosphopeptides (82% of total phosphopeptides) that showed a difference within a 0.2 ratio window in the comparison of CON and SC or CON-IL and SC-IL. Accordingly, correlated changes (r = 0.96) were observed in the significantly regulated proteins/phosphopeptides (adjusted *p*-value < 0.05) between the two comparisons (CON/CON-IL and SC/SC-IL), providing high confidence for our proteomics and phosphoproteomics measurements. Here, we identified 41 proteins and 84 phosphopeptides that were significantly increased and 109 proteins and 133 phosphopeptides that were significantly decreased in both CON and SC when compared to CON-IL and SC-IL (Table S1). In our data, most regulation was linked to apoptotic signaling generated in response to IL-1β exposure in β-cells as we expected (Table S2), since the IL-1β-mediated NF-κB inflammatory pathway plays crucial roles in cytokine-mediated β-cell toxicity and likely also in the pathogenesis of diabetes [14,21,22,23].

**Figure 3:**
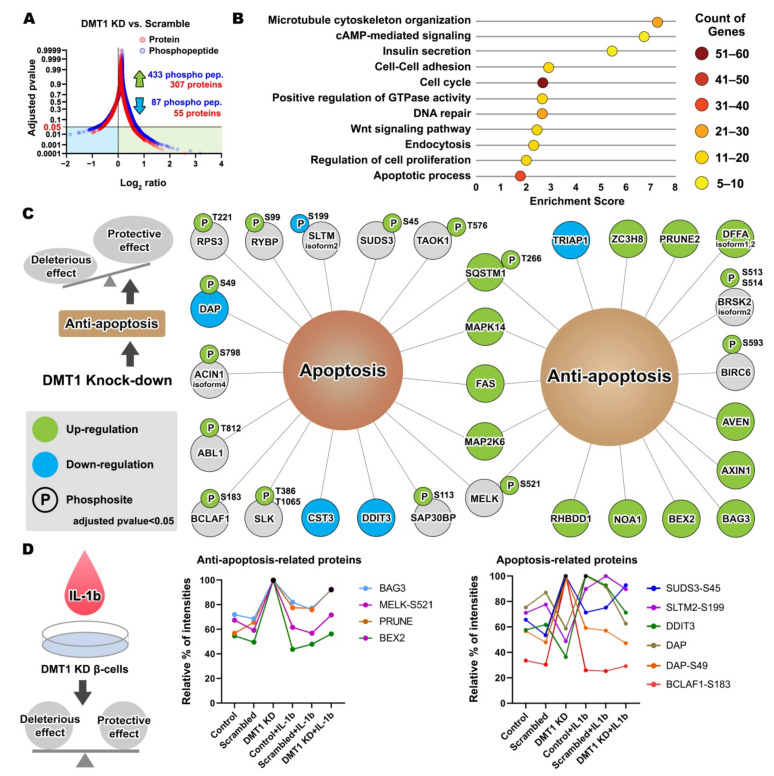
*DMT1* knock-down-mediated anti-apoptosis signaling and potential protective effect (**A**) Plot chart showing the increased 307 proteins and 433 phosphopeptides and decreased 55 proteins and 87 phosphopeptides in the comparison of *DMT1*-KD and SC. (adjusted *p*-value < 0.05) (**B**) Enriched biological processes for significantly regulated proteins and protein phosphorylation in the comparison of *DMT1*-KD and SC. DAVID Gene Ontology enrichment analysis showing the fold enrichment score for the indicated dots (*x*-axis) as well as the number of proteins assigned to classified functions (colors on circle). Corrected *p*-value < 0.05. (**C**) The relationship of the *DMT1* knock-down-mediated proteins and protein phosphorylation in the apoptosis and anti-apoptosis. P, phosphosite. (green: up-regulation, blue: down-regulation, grey: not significantly regulated) (**D**) The balance of protective effect and deleterious effect in the IL-1β exposed *DMT1* knock-down β-cells. Anti-apoptosis-related proteins/phospho proteins (**left**). Apoptosis-related proteins/phospho proteins (**right**).

KD and SC (adjusted *p*-value < 0.05; Figure 3A and Table S3).

### 2.2. IL-1β Activates Inflammation and β-Cell Death through Pro-Apoptotic Signaling Pathways

To investigate canonical signaling pathways controlling the IL-1β-induced (phospho)proteins, we conducted pathway analysis using the software Metacore. We identified several signaling pathways involved in IL-1β signaling. The top 10 signaling pathway categories are illustrated in Figure 2C, in the order of probability score (the sum of −log_10_ FDR acquired from proteomic and phosphoproteomic data), such as the IL-1 signaling pathway, NF-κB activation pathways, γ-secretase proteolytic targets, nerve growth factor (NGF) activation of NF-κB, Toll-like receptor (TLR) signaling pathway, and TNF-alpha and IL-1β induced hyperglycemia. The annotated genes under each signaling pathway are listed in Table S2. We also examined the contribution (−log_10_ FDR) in each signaling pathway that came from the proteome (beige bar in Figure 2C) and the phosphoproteome (yellow bar in Figure 2C).

In Figure 2D, we further analyzed protein-protein interaction networks for the regulated (phospho)proteins using the STRING tool and visualized the relationship among IL-1β-regulated proteins and protein phosphorylation. Specifically, we identified 16 new phosphosites in the IL-1β-induced proteins (NFKB2-S222, RIPK2-S176-S364, NGFR-T294-S306-S314, NOS2-S37, ADAM10-S83, SNW1-S224-S232, HN1-S82-S126, DLL1-S662, MX2-T12, and MAP3K7-T448-S459) in the Uniprot database that could be involved in NF-κB activation pathways, inflammatory responses, and ROS production according to the Metacore GO database. In the above NF-κB activation-associated proteins network, the regulated genes were mostly increased at the protein and phosphorylation levels after exposure to IL-1β, relevant for the apoptotic or anti-apoptotic signaling in β-cells (Figure 2D). A previous study showed that TNF induced cell adhesion disruption via the mixed lineage kinase domain-like-mediated activation of disintegrin and metalloproteinase domain-containing proteins (i.e., ADAM10 and ADAM17) together with ADAM substrates on the cell surface of HT29 cells [24], resulting in apoptosis, necroptosis, and inflammation [24,25]. Although the role of α- and γ-secretase is poorly understood in pancreatic β-cells, the NF-κB activation pathway has been found to increase the α- and γ-secretase proteolytic processes [targets: amyloid precursor protein (APP) and IL-1R2] in the pathogenesis of Alzheimer’s disease in the brain [26,27]. Similar to the proteolytic APP processing leading to the toxic amyloid-β formation, islet amyloid polypeptide (IAPP) amyloidosis and oligomer formation can cause oxidative stress, inflammation, and apoptosis through TLR-dependent NF-κB activation in islet β-cells [28,29]. Further, the interplay between the NOTCH signaling and the NF-κB-linked inflammatory pathway has been suggested to co-occur in the immune system by Rel/NF-κB-dependent *JAG1* expression in U266B cells [30]. In addition, the interaction between the NF-κB and NOTCH signaling pathways reduces the expression of anti-inflammatory genes such as *PPARγ* [31]. Interestingly, the inhibition of NOTCH signaling improves hepatic insulin resistance [32,33], triglyceride accumulation [34], and glucose tolerance during high-fat diet feeding in pancreatic β-cells [35]. Of note, ADAM10 and ADAM17 have been reported to regulate NOTCH cleavage and proteolysis, thereby triggering the activation of NOTCH/NOTCH-ligand (e.g., DLL1) signaling [36] that intervene in cell fate decisions [37]. Likewise, the relationship between the NF-κB activation pathway, NOTCH signaling, and α- and γ-secretase proteolytic processing could be an important initial molecular event resulting from the IL-1β mediated inflammatory response.

We observed that IL-1β exposure evoke the expression of IL-1R1 that may cause the activation of STATs (e.g., STAT1 and STAT3 with phosphorylation at Y705 and S727) as signal transducers and activators of transcription with other inflammatory responses, like NF-κB and NOTCH through STAT3 dimerization, nuclear translocation, and DNA binding [38,39]. STAT1 has been implicated in the onset of the autoimmune T1D [40]. In accordance with STAT3 activity, we observed significantly elevated proteins including transcription factors related to NF-κB activation pathway following the IL-1β mediated inflammatory attack in normal physiological β-cells (Figure 2D). In addition, NADH dehydrogenase 1 alpha subcomplex subunit 13 (NDUFA13) was decreased in IL-1β exposed β-cells, which is suggested to induce an electron leak from the mitochondria and STAT3 dimerization, triggering ROS generation and apoptosis in *NDUFA13* KO mice upon cytokine exposure [41]. Remarkably, we found nine transcription factors (JUN-S63, JUNB, SP1-T682, GTF2F1-S433, GTF2I-S655, DDIT3, TLE3-S217, NFKB1, and NFKB2-S222) cooperation with STAT1/3 that were significantly modulated in IL-1β exposed β-cells (Figure 2D). The interaction of activated STAT3 and NF-κB can synergistically mediate the transcription of pro-apoptotic and anti-apoptotic genes on cell viability [42,43]. For example, the myeloid differentiation primary response protein (MYD88) decreased in IL-1β exposed β-cells, which is related to the TLR pathway as a homeostatic effect in normal β-cells [44]. In contrast, MYD88-dependent TLR signaling has been shown to play a critical role in the innate or adaptive immune system via activation of IRAK families and NF-κB [45]. In this study, the expression of MYD88 and IRAK1 were significantly decreased. These results suggest that decreased MYD88 and IRAK1 levels might be independently involved in the immune system and adaptive flexibility of β-cells in the TLR pathway to support the maintenance of cell homeostasis against an excessive inflammatory response as negative feedback via tuning off inflammatory mediators. Moreover, other forms of interaction between transcription factors (e.g., STAT1 and SP1, STAT3 and JUN/JUNB) play pivotal roles in controlling the transcription of pro-survival and pro-death signals, thereby leading to IL-1β-activated transcription and inflammatory response [46,47,48]. For instance, the interaction between STAT1 and SP1 controls the transcriptional activation of intercellular adhesion molecule 1 (ICAM1) in response to IFN-γ [46]. In this study, ICAM1 was significantly increased in IL-1β-exposed β-cells, which is implicated in the pathogenesis of T1D [49,50]. Notwithstanding, it remains to be determined how IL-1β transduces these complex signals of interconnected cascades from IL-1 receptor to a transcriptional response in pancreatic β-cells needs further investigation, especially at the protein phosphorylation level.

### 2.3. DMT1 Knock-Down Promotes Anti-Apoptotic Signaling in β-Cells

We next investigated whether proteins and protein phosphorylation were significantly modulated by *DMT1*-KD, with and without exposure to IL-1β for 24 h. The unchallenged *DMT1*-KD cells were clearly distinguishable from the five other experimental groups in both the averaged proteome and averaged phosphoproteome data by a PCA plot (Figure 2A,B). We identified several significantly regulated proteins and phosphosites (362 proteins and 520 phosphopeptides) in the comparison of unchallenged *DMT1*-The functional annotation of the regulated proteins and protein phosphorylation was visualized using a plot chart (fold enrichment score, *x*-axis; number of genes, colors on circle) combined with GO biological process analysis (FDR < 0.05; Figure 3B). The GO biological analysis indicated that *DMT1* KD extensively affects important biological processes in β-cells, such as cAMP-mediated signaling, microtubule cytoskeleton organization, insulin secretion, endocytosis, apoptosis, cell cycle, Wnt signaling, regulation of cell proliferation, regulation of GTPase activity, cell-cell adhesion, and DNA repair (Figure 3B). The annotated genes under each GO biological process are listed in Table S4.

Notably, we identified anti-apoptotic or apoptotic modulators induced by the silencing of *DMT1* (Figure 3C). In the pro-apoptotic regulators, three proteins (CST3, DAP, and DDIT3) and 11 phosphoproteins (ABL1-T812, AXIN1 isoform 4-S798, BCLAF1-S183, DAP-S49, RPS3-T221, RYBP-S99, SAP30BP-S113, SLK-T386, T1065, SLTM isoform 2-S199, SUDS3-S45, and TAOK1-T576) were significantly increased, mostly at the phosphorylation level in the comparison of *DMT1*-KD and SC. In relation to the modulators of anti-apoptotic signaling, the production of 11 proteins (AVEN, AXIN1, BAG3, BEX2, DFFA-isoform 2, NOA1, PRUNE2, RHBDD1, SQSTM1, TRIAP1, and ZC3H8) and three phosphoproteins (BIRC6-S593, BRSK2 isoform 2-S513,S514, and SQSTM1-T266) were mostly increased in *DMT1*-KD versus SC (Figure 3C). All “classified genes” listed were confirmed for the appropriate aspect of apoptosis and anti-apoptosis from the literature (Table S5). Of anti-apoptotic mediators, six proteins (PRUNE2, AVEN, RHBDD1, ZC3H8, NOA1, and AXIN1) have been suggested to prevent the activation of apoptosis or inflammation. For instance, increased production of protein prune homolog 2 (PRUNE2) has been proposed to promote anti-apoptosis in pancreatic β-cells and was decreased in islets from patients with T1D [51]. Apoptosis and the caspase activation inhibitor (AVEN) inhibit APAF-1-mediated proteolytic activation of caspases in the cell death pathway, thereby suppressing apoptosis [52]. Over-induction of rhomboid-related protein 4 (RHBDD1) decreases Bcl-2-interacting killer-mediated apoptotic activity as a serine-type endopeptidase [53]. The gene mutation of zinc finger CCCH domain-containing protein 8 (ZC3H8) reduces the NF-κB–mediated inflammatory response as a repressor of inflammation [54]. The absence of nitric oxide-associated protein 1 (NOA1) causes oxidative stress through lack of complex IV stability, including consequent cell death, whereas induction of *NOA1* promotes reduction of oxidative stress via facilitating respiratory supercomplex formation [55]. Notably, Axis inhibition protein 1 (AXIN1) is a negative modulator of Wnt signaling [56], and loss of *AXIN1* can lead to apoptosis [57]. In islet β-cells, activation of Wnt signaling leads to the enhancement of cell proliferation by cell cycle modulators [58], and suppressed *AXIN1* impairs islet β-cell expansion, mass, and glucose tolerance [58].

To summarize the data so far, the *DMT1*-silencing regulated proteins identified are involved in the crosstalk between proteolytic processing, cell cycle arrest, Wnt signaling, and apoptosis, which could be emerging features of β-cell survival and crucial to balance the regulation of apoptosis [59]. Thus, our results provide previously uncharacterized evidence of a signaling mechanism governing inflammatory induced β-cell death or *DMT1* deficiency-mediated survival in physiologic or pathophysiologic conditions demanding β-cell resilience. Further, our data indicate that *DMT1* silencing could promote anti-apoptotic signal activators and suppress apoptotic transducers, resulting in improved β-cell function and survival by restraining multiple signaling pathways linked to DMT1-dependent signal transduction.

### 2.4. DMT1 Silencing Modulates the Balance between Apoptosis and Anti-Apoptosis Following Decreased Autophagic Flux in IL-1β Exposed β-Cells

We next explored which *DMT1* silencing-induced (phospho)proteins were associated with the switch from a pro- to an anti-apoptotic response in IL-1β exposed β-cells (Table 1 and Table S6). Of these, nine (phospho)proteins (BAG3, BCLAF-S183, BEX2, DAP-S49, DDIT3, MELK-S521, PRUNE2, SLTM isoform 2-S199, and SUDS3-S45) were clearly reverted to the normal state after IL-1β exposure (Figure 3D). Interestingly, the *DMT1* KD-regulated (phospho)proteins were involved in the regulation of apoptotic signaling pathway in the GO database, and the anti-apoptotic (phospho)proteins were mainly associated with autophagy in β-cells (Table 1). As mentioned, pro-inflammatory cytokines released from islet-resident inflammatory cells can facilitate β-cell iron import by up-regulating *DMT1* expression, leading to excessive ROS production, and thus autophagy and ultimately cell death [1,60]. A protective mechanism of autophagy helps to prevent apoptosis and pro-apoptotic signals by clearing damaged organelles and proteins [61,62]. However, ROS-induced aberrant autophagy and ferroptosis can also be involved in mechanisms of cell demise [63,64,65]. Further, excess iron-induced DMT1 production is a key player in ferroptotic cell death [66]. Iron overload by DMT1 induction can promote autophagy and apoptosis in osteoblasts [67]. Remarkably, *DMT1* KD restored autophagy regulators which have a very important protective role in cell survival and death (e.g., SQSTM1-T266, PIK3C3-S243, RNF185-T106, BAG3, DAP, and STING) in IL-1β exposed cells, [65,68,69]. For example, Bcl2-associated athanogene 3 (BAG3) facilitates selective macroautophagy or autophagy through ascent of glutamine uptake and glutaminolysis [70]. A decreased BAG3 has been shown to enhance apoptosis together with oxidative stress and attenuate cell survival [71]. In addition, maternal embryonic leucine zipper kinase (MELK) activity is essential in the regulation of apoptosis in conjunction with the activation of the NF-κB pathway via sequestosome 1 (SQSTM1) [72,73]. SQSTM1 is a multifunctional protein that is linked to numerous forms of cellular stress and accounts for the interplay between autophagy, ubiquitin-proteasome system (UPS), and DNA repair [68]. We found interactions between BAG3, MELK (phosphorylation at S521 by autocatalysis in IFN-γ-activated macrophages [69]), SQSTM1 (phosphorylation at T266), ICAM1, and IL1R1, all involved in autophagy leading to cell survival. A GWAS on T2D subjects identified a specific relationship with a polymorphism (rs5498) for a gene encoding a unique ICAM1 protein [74], which was previously shown to be up-regulated in patients with T2D [75] and diabetic nephropathy [76]. Death-associated protein (DAP) is also known as a mediator of apoptosis and an inhibitor of autophagy through the inhibition of mTOR-dependent phosphorylation [77,78,79]. After reduction of *DMT1* expression, DAP protein production was decreased, whereas the phosphorylation of DAP at S48 was increased, which may prevent apoptosis.

Furthermore, we identified dephosphorylation of E3 ubiquitin-protein ligase (RNF185) at T106 (new phosphosite) upon β-cell inflammation, and the phosphorylation level was recovered to normal levels by *DMT1* silencing. RNF185 is responsible for the ER-associated degradation (ERAD) pathway [80,81] and selective mitochondrial autophagy [82]. ER stressors (thapsigargin and tunicamycin) provoked significant expression of RNF185 which is shown significant resistance to ER stress in an E3 activity-dependent manner [81]. The role of phosphorylation at T106 is currently unknown, but our results suggest that an elevated phosphorylation of RNF185 at T106 might be a positive modulator in the ERAD pathway upon inflammatory attack, thereby mitigating ER stress. Interestingly, the protein STING (stimulator of interferon genes, also known as TMEM173) has been suggested to activate multiple functions in the innate immune response, autophagy and induction of NF-κB, IFN, and IRF3-dependent genes [83,84,85]. Consistent with these findings, STING and NF-κB were increased in response to IL-1β but were decreased by *DMT1* silencing.

Taken together, we provide evidence for previously unrecognized interplay between protein degradation pathways relevant in the modulation of autophagy compromising cell viability. Therefore, the inhibition of *DMT1* may profit from β-cell replenishment to inflammatory stress through the prevention of ROS and autophagy.

**Figure 4:**
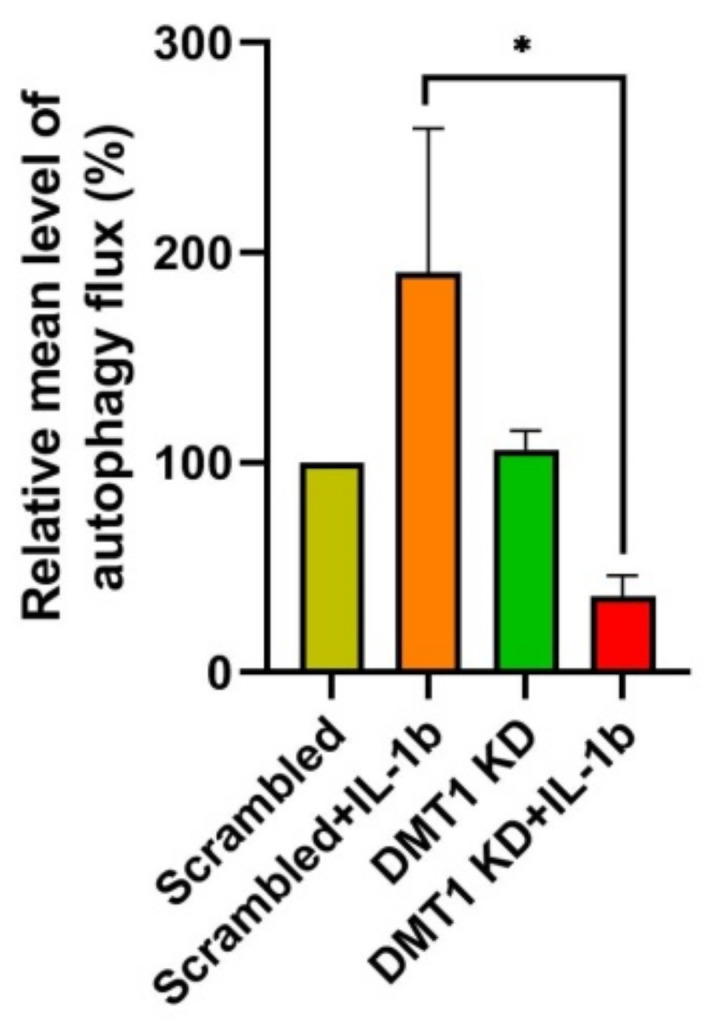
Quantification of autophagic activity using fluorescence spectrophotometric assay. The mean of relative fluorescence intensity was determined using CYTO-ID autophagy detection kit (Enzo life sciences), * *p* < 0.05, *n* = 3 biological replicates.

To validate the functional relevance of these key novel findings coming out of our integrated proteomics strategy, we next investigated autophagic activity by the autophagy CYTO-ID assay that quantifies autophagic vacuoles and thus autophagic flux. Interestingly, the autophagic flux was significantly decreased in *DMT1*-KD-IL when compared to SC-IL, whereas the autophagic activity was increased by IL-1β exposure in SC control cells (Figure 4). Although the mechanism of autophagy-dependent cell death remains to be elucidated, our results suggest that the regulation of autophagic activity might be important for the relationship between iron overload and cell demise to support the maintenance of β-cell homeostasis against an inflammatory assault.

### 2.5. DMT1 Silencing Reverts the IL-1β-Induced Responses towards the Normal State

**Figure 5:**
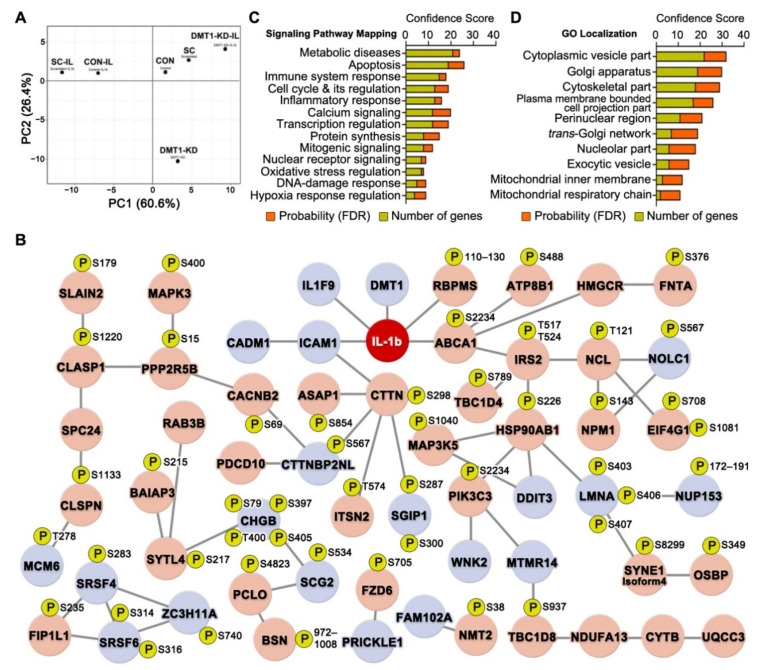
Replenishment of potential protective proteins and protein phosphorylation in IL-1β exposed *DMT1* knock-down β-cells. (**A**) PCA plots of the proteome and phosphoproteome profiles of Control (CON), Scrambled (SC), *DMT1* KD (*DMT1*-KD), Control + IL-1β (CON-IL), Scrambled + IL-1b (SI), *DMT1* KD + IL-1β (*DMT1*-KD-IL) cells. (**B**) β-cell inflammation-protective proteins interaction network (PPIN) among potential protective proteins and/or phosphorylation in IL-1β exposed *DMT1* depletion β-cells. Red node: significantly increased levels in both SC/SI and *DMT1*-KD-IL/SI. Blue node: significantly decreased levels in both SC/SI and *DMT1*-KD-IL/SI. P: phosphosite. (**C**) Mapping of signaling pathways of potential protective proteins and/or phosphorylation formed prominent in order by confidence score (sum of FDR and genes). (**D**) Gene Ontology subcellular localization of potential protective proteins and/or phosphorylation formed prominent in order by confidence score (sum of FDR and genes).

To elucidate underlying mechanisms of the protective response caused by reduction of *DMT1* expression against the adverse β-cell responses to inflammation, we next investigated anti-inflammatory and cell protective (phospho)proteins in *DMT1*-KD-IL (compared to CON-IL and SC-IL; adjusted *p*-value < 0.05 in both SC/SC-IL and *DMT1*-KD-IL/SC-IL). We identified 38 proteins and 63 phosphopeptides which were restored by *DMT1* silencing (30~50% less than SC-IL) after exposure to IL-1β (Table S6), visualized using a plot chart (Figure 5A). In the PCA analysis, the *DMT1*-KD-IL cluster was close to the control cell clusters (CON and SC), but distinct from CON-IL and SC-IL clusters in both the proteome and phosphoproteome analyses (Figure 5A). Notably, the *DMT1*-KD-IL clusters were remote from the other clusters the PCA plot (Figure 5A).

To explore the interrelations between the protective (phospho)proteins, we employed a combination of STRING (protein-protein interaction) and Metacore (signaling pathway) analyses. In the protective protein interaction network (PPIN), 59 genes (Up-regulation: 39, Down-regulation: 20) were tightly interconnected. Of these genes, five (DMT1, IL1F9, ICAM1, RBPMS, and ABCA1) were closely linked to the IL-1β protein (Figure 5B). A meta-analysis of genome-wide association studies (GWAS) identified IL-1β-specific association signals with a polymorphism (rs11677903) of a gene encoding a unique IL1F9 (also known as IL36G) protein [86]. Further, IL1F9 has been suggested to activate NF-κB, MAPKs, and JNK, resulting in enhanced inflammatory responses [87]. In addition, the RNA-binding protein with multiple splicing (RBPMS), previously uncharacterized in pancreatic β-cells, is known to be up-regulated by pro-inflammatory cytokines like TGF-β and IL-1β [88,89]. Two previous GWAS meta-analyses identified T2D-associated polymorphism (rs9282541 and rs1800977) of a gene encoding a unique ABCA1 [90,91], involved in insulin secretion, glucose tolerance, and cholesterol homeostasis [92,93,94,95].

We next investigated the signaling pathways and subcellular components of the PPIN using Metacore. Notably, these were involved in apoptosis, metabolic diseases, calcium signaling, transcription regulation, cell cycle and its regulation, and immune system response (Figure 5C). In addition, GO subcellular localization showed that the proteins were associated with the cytoplasmic vesicle compartment, Golgi apparatus, trans-Golgi network, and plasma membrane-bounded cell projection part (Figure 5D). Interestingly, these cellular organelles are well known as key components of the secretory pathway in the cell. Improved secretory functions may contribute to beneficial outcomes that promote cell survival and resistance to cell demise, but not exhibited in over-activation of the secretory pathway [96]. Hansen et al. reported that *DMT1* knock-out islets enhance glucose-stimulated insulin secretion (GSIS) when compared to IL-1β exposed islets [1]. In addition, β-cell-specific *DMT1* knock-out improves glucose tolerance and circulating insulin levels in T1D and T2D models such as multiple low-dose of streptozotocin and high-fat diet mice [1]. In our results (Figure 5B and Table S6), eleven (phospho)proteins (HMGCR, IRS2-T517, T524, PCLO-S4823, SYTL4-S217, BAIAP3-S215, RAB3B, ABCA1-S2234, CLASP1-S1220, CADM1, SCG2-S534, and CHGB-S79,S397,T400,S405) were involved in insulin secretion, and they were restored towards normal in *DMT1*-silenced β-cells against IL-1β exposure. For instance, β-cell-specific silencing of 3-hydroxy-3-methylglutaryl-coenzyme A reductase (HMGCR) acutely triggered the reduction of β-cell mass and insulin secretion after postnatal day 9 in mice and eventually leading to the development of diabetes [97]. Knock-down of cell adhesion molecule 1 (CADM1) promotes GSIS in rat and human islet β-cells whereas the induction of *CADM1* inhibits GSIS [98]. Protein piccolo (PCLO) and Ras-related protein Rab-3B (RAB3B) are involved in the vesicle docking and fusion to the plasma membrane for insulin secretion from β-cells [99]. Here, we provided highly reliable localization of the phosphosites on proteins that are involved in insulin secretion, but the role of phosphorylation is currently unknown. More research is needed to understand the functions for phosphorylation of insulin secretion-associated proteins identified by our phosphoproteomics experiment. Collectively, our data support that *DMT1* silencing may lead to a beneficial way of improving insulin secretion from β-cells upon exposure to pro-inflammatory cytokine, as previously described [1].

### 2.6. Validation of Phosphoproteins Potentially Protective against β-Cell Inflammatory Response Using Parallel Reaction Monitoring (PRM)

We observed five novel phosphosites of the proteins (LRRFIP1, LSR, TBC1D4, NOLC1, and NCL) based on the phosphoproteomics data that are associated with the regulation of oxidative stress. The phosphopeptides were validated in control and *DMT1*-silenced β-cells, with or without IL-1β exposure using the PRM approach [16]. They were selected based on the following criteria: being unique peptides, the most repeatable charge states corresponding to intensity, and being less than 20 amino acids. We excluded possible modifications, but phosphorylation was included on Ser or Thr. Synthetic heavy isotope-labeled phosphopeptides were generated and used for internal quantitation standards for PRM validation of all four sample groups (Figure S1A). Intact phosphopeptides and heavy-labelled phosphopeptides co-eluted in the LC-MS/MS analysis, allowing accurate quantitation (Figure S1B). The phosphopeptide sequences were validated by MS/MS spectra acquisition which allowed precise phosphosite localization (Figure S1B). We confirmed a high correlation for LC retention time (Pearson correlation of 0.999) between intact and heavy peptides in all groups.

By using the PRM strategy, we confirmed the levels of the five endogenous phosphopeptides, which correlated with the initial TMT quantitation (Figure S1C). The phosphorylation levels of LRRFIP1-T539, LSR-T449, NOLC1-S567 were increased in response to IL-1β exposure in β-cells, and they were restored towards normal in *DMT1*-silenced β-cells (Figure S1C). In contrast, control cells’ exposure to IL-1β lowered the phosphorylation levels of TBC1D4-S789 and NCL-T121, but *DMT1* silencing (30~50% less than SC-IL) resulted in higher phosphorylation compared with the control, which could not be diminished by IL-1β exposure (Figure S1C).

### 2.7. The Protective Protein Interaction Network Reveals the Alteration of New Phosphoproteins in Relation to β-Cell Anti-Inflammation and Anti-Apoptosis

**Figure 6:**
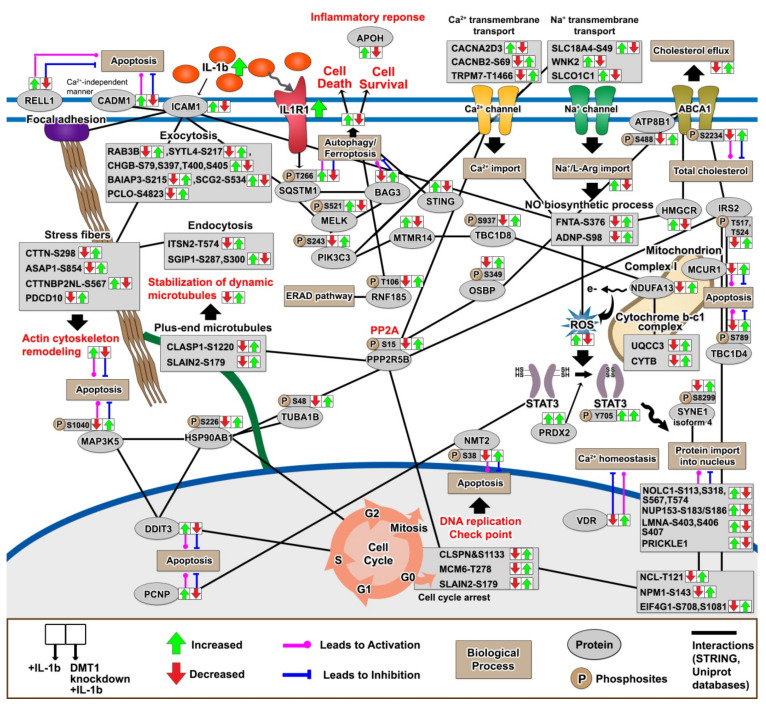
Schematic overview of potential protective proteins associated with anti-apoptosis or anti-inflammation in *DMT1*-silenced β-cells against pro-inflammatory attack. (Green: significantly increased, Red: significantly decreased).

Finally, we characterized the signaling pathways relevant to the protection against IL-1β-induced changes in DMT1-silenced β-cells to understand how amelioration of pro-apoptotic signaling networks contributes to β-cell survival. In particular, we identified 16 new phosphosites in the potential protective proteins (TBC1D4-S789, TBC1D8-S937, CHGB-S79-T400-S405, BAIAP3-S215, PCLO-S4823, CTTN-S298, FNTA-S376, RNF185-T106, PPP2R5B-S15, PIK3C3-S243, CACNB2-S69, ABCA1-S2234, ATP8B1-S488, and SYNE-isoform 4-S8299) in the Uniprot database, that were involved in the regulation of autophagy, apoptosis, actin cytoskeleton remodeling, stabilization of dynamic microtubules, ER-associated degradation (ERAD) pathway, Ca^2+^/Na^+^ transmembrane transport, cholesterol efflux, ROS production, and cell cycle arrest, but the functional roles of specific phosphosites on these proteins are presently unknown (Figure 6). Recent studies have reported that autophagy-dependent ferroptosis increases iron accumulation or lipid peroxidation by selective degradation of pro-survival proteins [63,100,101,102,103]. In addition, iron overload triggers the activation of Na^+^/K^+^ transmembrane transport which may lead to autophagy-dependent ferroptosis [104,105]. Although the primary function of NF-kB and MAPK are well defined, we provided previously unknown potentially protective proteins induced by *DMT1* silencing (e.g., STING and MCUR1), and these potentially protective proteins formed a signaling network based on protein-protein interaction analysis with a high confidence score (0.7) in the STRING database (Figure 6).

We highlight the relationship between the cell cycle arrest and degradation pathways, which could be critical for β-cell homeostatic effect beyond the activation of an islet-immune cascade. Remarkably, we observed the restoration of cell cycle arrest modulators after *DMT1* silencing during β-cell inflammation, as indicated by the reversed regulation presented by arrows in Figure 6. In the cell cycle progression, the checkpoints are induced to repair DNA damage, with apoptosis following permanent DNA damage [106]. For instance, DNA-damage inducible transcript 3 (DDIT3, also known as CHOP) is implicated in ER stress, triggering pancreatic β-cell apoptosis via inducing G_1_ cell cycle arrest as observed in T2D [107]. The suppression of the kinetochore protein (SPC24) activates G_1_ cell cycle arrest and apoptosis in cancer cells [108,109]. In addition, BEX2 is suggested to act in the regulation of mitochondrial apoptosis and G_1_ cell cycle arrest [110,111]. BCL2-associated transcription factor 1 (BCLAF1) is known to be a regulator in NF-κB activation, triggering the cell cycle arrest associated with DNA damage [112]. Interestingly, we observed the new phosphosites of lamins (LMNA-S403, S406, S407, and LMNB1-S393) and transcription factor AP-1 (JUN-S49), which are dynamically modulated toward a protective effect induced by *DMT1* silencing (Figure 5). Nuclear lamins are well known to be involved in DNA replication, transcription and chromatin organization, and cell cycle arrest [113]. Prelamin-A/C (LMNA) and JUN can interact and co-localize at the nuclear envelope for the control of AP-1 transcriptional activity and cell cycle arrest [114]. *LMNA* deficiency in cells raises proliferation, whereas *LMNA* overexpression enhances cell cycle arrest [114]. Further, activated JUN contributes to apoptosis [115], and its activation is sustained by stimulating with pro-inflammatory cytokines, such as TNF-α or IL-1β [116]. In our data, the phosphorylation of lamin-B1 (LMNB1) was reduced in response to IL-1β, as opposed to the direction of LMNA and JUN. LMNB1 plays a critical role in ensuring normal cell proliferation and cell cycle progression [117,118]. Moreover, many proteins (i.e., CLSPN-S1133, MCM6-T278, SLAIN2-S179, and NMT2-S38) related to cell cycle arrest were modulated upon *DMT1* silencing at the phosphorylation level, which indicates a role of their phosphorylation in response to pro-inflammatory cytokines and the balance between apoptosis and cell proliferation in the regulation of cell cycle arrest is currently unknown. Nonetheless, we hypothesize that our findings could shed light on key factors to counterbalance deleterious effect in the control of cell cycle arrest by silencing *DMT1*.

## 3. Materials and Methods

### 3.1. Reagents

All chemicals were of the highest purity obtainable and purchased from Sigma (Sigma-Aldrich, St. Louis, MO, USA) unless stated otherwise.

### 3.2. Cell Culture

About 2 × 10^6^ rat insulin-producing β-cells (INS-1E) were seeded on 10 cm non-coated Petri dishes (Nunc^TM^, Roskilde, Denmark) and cultured in RPMI-1640 with Glutamax (Gibco^®^, Paisley, Scotland, UK) supplemented with 5% fetal bovine serum (Biowest, Nuaillé, France), 100 U/mL penicillin and 100 µg/mL streptomycin (Gibco^®^, Paisley, Scotland, UK), 1% sodium pyruvate, and 0.1% β-mercaptoethanol (complete medium, CM) at 37 °C and 5% CO_2_. After three days, the medium was changed to Opti-MEM^TM^ reduced serum medium with Glutamax (Gibco^®^, Paisley, Scotland, UK), and cells were cultured for 24 h before siRNA transfection. Three different siRNAs targeting *DMT1*, scrambled siRNA (Qiagen, Germantown, MD, USA), or DharmaFECT transfection reagent (Dharmacon, Lafayette, Indiana, CO, USA) were diluted separately in Opti-MEM^TM^ at room temperature (RT) for 5 min (30 nM final concentration). The diluted siRNAs were incubated with diluted DharmaFECT at RT for 20 min. After 24 h of transfection, the medium was replaced by CM and cells cultured overnight for recovery. The medium was replaced, and then INS-1E cells were exposed to 50 pg/mL of recombinant rat IL-1β (R&D system, Minneapolis, MD, USA). After 24 h incubation INS-1E were harvested.

### 3.3. mRNA Extraction

Cells were lysed directly in the culture dish using RNAzol^®^ RT (Sigma) after removing culture medium. The lysate was transferred to a 1.5 mL tube and RNases-free OmniPur water (Sigma, Darmstadt, Germany) was added. The solution was vigorously mixed for 15 s, incubated for 15 min at RT, and subsequently centrifuged at 12,000× *g* for 15 min at RT. The supernatant (containing RNA) was transferred to a new tube, and the pellet (containing DNA, proteins, and polysaccharides) was immediately stored at −20 °C. Ethanol (75% *v*/*v*) was added to the supernatant and the mixture incubated for 10 min at RT to precipitate the mRNA. The tube was centrifuged at 12,000× *g* for 8 min at RT. Eighty-five % of the supernatant was removed and washed first with 75% ethanol (Sigma) and then with 70% isopropanol (Sigma, *v*/*v*) for mRNA precipitation. The supernatant was removed by centrifugation at 8000× *g* for 3 min at RT. The isolated mRNA was solubilized in RNase-free water (Sigma, Darmstadt, Germany) at a concentration of 1–2 μg/μL. The mixture was vigorously vortexed for 5 min at RT. The mRNA concentration (OD 260/280 ratio greater than 1.9) was measured using a NanoVue^TM^ plus spectrophotometer (GE healthcare, Freiburg, Germany), and the mRNA was stored at −80 °C.

### 3.4. Real-Time PCR

Complementary DNA (cDNA) was synthesized with a high-capacity cDNA reverse transcription kit (Applied Biosystems, Foster City, CA, USA) following the manufacturer’s protocol. For *DMT1* amplification, the Rn_Slc11a2_1_SG QuantiTect Primer Assay (QT00182623, Qiagen, Germantown, MD, USA) was used and analyzed with the FastStart Essential DNA Green Master Mix (Roche, Hvidovre, Denmark) using the LightCycler^®^ 96 system (Roche, Basel, Switzerland). The expression level was normalized to the *β-actin* mRNA level (QT00193473, Qiagen, Germantown, MD, USA).

### 3.5. Western Blot Analysis

Protein extracts of control or IL-1β exposed (0, 50, and 100 pg/mL) INS-1E cells were added protein loading buffer (10% glycerol, 10% sodium dodecyl sulfate, 5% β-mercaptoethanol, 0.05% bromophenol blue, and 0.5 M Tris HCl, pH 6.8), and the solutions were heated at 95 °C for 5 min. Proteins were then separated using a Bis-Tris 4–12% gel (Invitrogen, Carlsbad, CA, USA) and transferred onto a Hybond ECL nitrocellulose membrane (GE Healthcare, Hatfield, UK) using a Trans-Blot SD cell (Bio-Rad, Hercules, CA, USA). After transfer, the membranes were incubated for 1 h with 5% skimmed milk powder in 50 mM Tris–HCl (pH 7.4) and 150 mM NaCl (TBS, Sigma) at RT to prevent nonspecific binding. Blots were then incubated overnight with primary antibodies [rabbit polyclonal anti-DMT1 (62 kDa, 1:1000, NRAMP24-A, Alpha Diagnostic International Inc., San Antonio, TX, USA) and mouse monoclonal anti-β-actin (43 kDa, 1:1000, sc-47778, Santa Cruz, Dallas, TX, USA)] and diluted in 5% skimmed milk powder in TBS at 4 °C. After three washes for 10 min in 0.1% Tween–TBS, a secondary antibody was added at RT using an anti-rabbit IgG peroxidase conjugate (1:5000, #7074, Cell Signaling, Beverly, MA, USA) or an anti-mouse IgG peroxidase conjugate (1:5000, ab6728, Abcam, Cambridge, MA, USA) in a solution containing 3% skimmed milk powder and 0.1% Tween–TBS. After three washes, bands were visualized using ECL detection reagent and Amersham^TM^ Imager (GE Healthcare). Bands were quantified by densitometric analysis using ImageJ software (https://imagej.nih.gov/ij/, accessed on 9 November 2018). For relative quantification, DMT1 band intensity from the respective IL-1β exposed INS-1E cells was normalized to β-actin using the ImageJ.

### 3.6. Fluorescence Spectrophotometric Assay for Autophagic Flux

The Enzo CYTO-ID autophagy detection kit (Enzo Life Sciences, Farmingdale, NY, USA) was employed following the manufacturer’s instructions. Briefly, 2 × 10^4^ INS-1E cells were seeded on a 96-well plate (Nunc^TM^, Roskilde, Denmark). After *DMT1* siRNA transfection, the cells were treated with or without 50 pg/mL of recombinant rat IL-1β for 24 h. After washing with the buffer provided with the kit, the kit fluorescence buffer was added for 30 min at 37 °C in the dark. The fluorescence intensity (FITC: emiision~530 nM excitation~480 nM and Hoechst: emiision~480 nM excitation~340 nM) was detected using FLUOstar^®^ Omega plate reader (BMG Labtech, Aylesbury, UK).

### 3.7. Protein Extraction, Sample Preparation, and TMT Labeling

The pellet from the RNA extraction (containing DNA, proteins, and polysaccharides) was solubilized with 4 volumes of chilled acetone and then incubated overnight at −20 °C for precipitation. The tube was centrifuged at 12,000× *g* for 10 min at 4 °C. The supernatant was removed, and the pellet was dissolved in denaturation buffer [0.3 M guanidine hydrochloride in 95% ethanol (*v*/*v*), 2.5% glycerol, and phosphatase/protease inhibitor cocktail (Roche, Basel, Switzerland)] for washing and solubilization of hydrophobic proteins [119]. The sample was incubated for 10 min at RT and then centrifuged at 8000× *g* for 5 min at 4 °C. The supernatant was removed, and the pellet was washed twice with guanidine hydrochloride-denaturation buffer and finally with ethanol supplemented with 2.5% glycerol and phosphatase/protease inhibitor cocktail without compromising the solubility of the pellet [120]. The mixture was incubated for 10 min at RT and then centrifuged. The supernatant was removed, and the pellet was solubilized in 50 mM HEPES [4-(2-hydroxyethyl)-1-piperazineethanesulfonic acid buffer, pH 8.5] containing 6M Urea, 2 M Thiourea, 10 mM Dithiothreitol (DTT), and phosphatase/protease inhibitor cocktail. The protein mixture was incubated for 2 h at RT, and then the sample was incubated with 20 mM iodoacetamide for 30 min in the dark. To completely remove phenol and guanidine thiocyanate, the sample was mixed in a mixture of methanol, chloroform, and distilled water (4:1:3, *v*/*v*/*v*), and then centrifuged at 10,000× *g* for 15 min at RT. The supernatant was removed, and the pellet was dissolved in 50 mM HEPES buffer (pH 8.5). Trypsin was added to the sample (1:50, *w*/*w*) and the solution was incubated overnight at RT. The digested peptide mixture was lyophilized before TMT labeling. A total of 100 µg of sample from each group were labelled with the TMT 11 plex kit (Thermo Scientific, San Jose, CA, USA) based on the quantification achieved from Qubit^®^ Fluorometric Quantitation (Thermo Scientific, Waltham, MA, USA) [TMT-6×biological three replicates: “Control” (126), “Scrambled” (127N or 127C), “*DMT1* KD” (128N or 128C), “Control + IL-1β” (129N or 129C), “Scrambled + IL-1β” (130N or 130C), and “*DMT1* KD + IL-1β” (131N or 131C)]. The TMT labeled peptides were mixed in equal ratios and analyzed simultaneously.

### 3.8. Enrichment of Phosphorylated Peptides

Phosphopeptide enrichment was performed from the multiplex labeled peptide mixture according to a previously published protocol [16,17,18]. The eluted phosphopeptides were passed over a C8 stage tip (3 M^TM^ Empore^TM^ Bioanalytical Technologies, St. Paul, MN, USA) to retain the titanium dioxide beads (TiO_2_; GL Science, Tokyo, Japan). The 50% acetonitrile wash of the C8 stage tip was collected together with the phosphopeptides, and they were dried by vacuum centrifugation to produce the enriched phosphopeptide fraction. The flow-through from the TiO_2_ enrichment step was dried by vacuum centrifugation to produce the nonmodified peptide fraction. The phosphopeptide or nonmodified peptide fraction was acidified (pH < 3) with trifluoroacetic acid (TFA) and desalted on a Poros Oligo R3 (PerSeptive Biosystems, Framingham, MA, USA) stage tip column and subsequently fractionated using high-pH reversed-phase (High-pH RP) fractionation as described in [17].

### 3.9. Liquid Chromatography-Tandem Mass Spectrometry Analysis

All fractions were dissolved in buffer A (0.1% formic acid; FA) and analyzed using an LC-MS/MS system consisting of an Easy-nLC and a Q-Exactive HF mass spectrometer (Thermo Scientific). The samples were loaded either directly onto a 2 cm trap column (100 μm inner diameter (ID)) and separated on a 20 cm fused silica capillary analytical column (75 µm ID) or separated directly on a 20 cm fused silica capillary column (75 μm ID). All columns were homemade and packed with ReproSil-Pur C18 AQ 3 μm reversed-phase material (Dr. Maisch, Ammerbuch-Entringen, Germany). The peptides were eluted using 70–142 min gradients from buffer B (95% Acetonitrile (ACN), 0.1% FA) and introduced into the MS instrument via nano-electrospray. A full MS scan in the mass area of 350–1400 Da was performed in the Orbitrap with a resolution of 60,000, an AGC target value of 3 × 10^6^, and a maximum injection time of 50 ms. For each full scan, the settings for the higher energy collision dissociation (HCD) were as follows: top 15 most intense precursor ions, AGC target value of 1 × 10^5^, maximum injection time of 100 ms, isolation window of 1.2 *m/z*, normalized collision energy of 32, and a dynamic exclusion window of 30 s.

### 3.10. Protein Identification and Quantification

The raw MS data sets were processed for protein identification using the Proteome Discoverer (PD, v2.3, Thermo Scientific) and the Sequest HT algorithm with a peptide mass tolerance of 10 ppm, fragment m/z tolerance of 0.05 Da, reporter ion m/z tolerance half-width of 2 mDa, and a false discovery rate (FDR) of 1% for proteins and peptides. All peak lists were searched against the UniProtKB/Swiss-Prot database (v2017-10-25, 34,352 entries) of rat and mouse sequences (mouse sequences are included as the rat protein database is not complete) using the following parameters: enzyme, trypsin; maximum missed cleavages, 2; fixed modification, carbamidomethylation (C), TMT tags (K, peptide N-termini); variable modifications, oxidation (M) and phosphorylation (S, T, Y). For relative protein quantification, the output file was exported into Microsoft Excel from PD. Then, protein relative expression values from the respective unique peptides (only in a single protein) were calculated by summing all peptide intensities of each protein and normalized to the number of the total intensity of each group to estimate the relative amounts of the different proteins within the sample. The resulting ratios were log-transformed (base = 2) to achieve a normal distribution, and then log_2_ ratios were averaged per unique protein or phosphopeptide for subsequent analysis. Three biological replicates were performed. All differentially expressed proteins and altered phosphopeptides were defined using statistical methodology (z-test for adjusted *p*-value < 0.05 with the Benjamini-Hochberg correction) from the overlapping proteins or phosphopeptides and the same expression direction (log_2_-fold changes: positive or negative) in all biological replicates. Phosphosite localization probability (at least 75%) was checked in the MS/MS data sets using PhosphoRS [20].

### 3.11. Bioinformatics Analysis of Proteomic and Phosphoproteomic Data

Gene Ontology (GO) annotation enrichment analysis was performed using DAVID Bioinformatics Resources (version 6.8) [121], and signaling pathway analysis was performed using Metacore (Clarivate analytics). Protein-protein interaction analysis was searched against the STRING database (version 11.0) [122]. Results were analyzed using confidence score (FDR and number of genes) and produced using GraphPad Prism 8 (GraphPad Software).

### 3.12. Validation of Selected Protein Phosphorylation Using Parallel-Reaction Monitoring (PRM) Assay

PRM has been used for exact quantification of targeted protein phosphorylation using stable-isotope-labeled peptides and LC-MS/MS approach [16]. A PRM assay was performed using five heavy isotope-labeled synthetic phosphopeptides (JPT Peptide Technologies) on a Q-Exactive™ HF-X mass spectrometer (Thermo Scientific) in two technical replicates. All heavy-labeled (on K or R) peptides (500 fmol) corresponding to tryptic phosphopeptides found in the TMT analysis [leucine-rich repeat flightless-interacting protein 1 (LRRFIP1-T539), lipolysis-stimulated lipoprotein receptor (LSR-T449), TBC1 domain family member 4 (TBC1D4-S789), nucleolar and coiled-body phosphoprotein 1 (NOLC1-S567), and nucleolin (NCL-T121)] were spiked into tryptic peptides originating from 1 μg of cell lysate proteins as internal standards. Subsequently, an equal amount of peptide mixture from each group was collected, and the phosphopeptides were enriched using TiO_2_ enrichment. The resulting phosphopeptides were desalted using Poros Oligo R3 RP micro-columns prior to PRM analysis. The samples were resuspended in 0.1% FA and loaded onto a 25 cm analytic column consisting of fused silica capillary (75 μm ID) packed with ReproSil-Pur C18 AQ 1.9 µm reversed-phase material (Dr. Maisch, Ammerbuch-Entringen, Germany) using an EASY-nLC system (Thermo Scientific). The peptides were eluted with an organic solvent gradient from 100% phase A (0.1% FA) to 32% phase B (95% ACN, 0.1% FA) at a constant flowrate of 300 nl/min. The PRM method consisted of a full MS scan configured as above, followed by targeted MS/MS scans for each selected phosphopeptide with a 3-min retention time window, as defined by a time-scheduled inclusion list. The parameters were set as follows: MS, 120K resolution; MS/MS, 15K resolution; 3e6 AGC target, 15 ms maximum injection, 1.1 m/z isolation window, and HCD of 27–28.

We used Skyline (version 20.1.0.28) for quantitative analysis of the generated PRM data [18]. Briefly, raw MS files were imported into Skyline. Then a FASTA file (from Uniprot) containing targeted proteins was imported to Skyline. We applied the highest confidence of dot-product score (≥0.95 which provides a correlation score between the measured product ion peak areas and the fragment ion intensities for the precursor ions. We compared the individual phosphopeptide amounts with the fragmentation ratio (intact light peak area/heavy standard peak area) in controls and *DMT1* KD with the presence or absence of IL-1β as assessed by PRM assay.

## 4. Conclusions

In insulin-producing β-cells, pro-inflammatory cytokine-mediated inflammation is contributing to cell death, eventually leading to the onset and progression of diabetes. Our results suggest molecular modulations on two aspects of apoptotic and anti-apoptotic signaling from the proteome and phosphoproteome data that may lead to the prevention of β-cell inflammation and the improvement of β-cell function in a *DMT1* genetic silencing cell model. After silencing *DMT1*, we found the replenishment of new phosphosites on many proteins which are associated with the early molecular event of β-cell destruction process by IL-1β that could be associated with cell survival. However, the exact function of these identified targets has not been investigated. Nevertheless, our global characterization of (phospho)proteins has provided in-depth molecular insights into important signaling pathways initiated in *DMT1* KD as a mechanism to protect against pro-inflammatory cytokine attack. The relationship between the signaling pathways provides potentially functional mechanisms governing anti-apoptotic signal activators and suppressing apoptotic transducers at the level of phosphorylation. Further in vivo study on an in-depth understanding of how *DMT1*-silencing-induced signaling pathways contribute to the β-cell survival is needed, and DMT1 inhibition might provide more cues for the development of therapeutic strategies in diabetes. We also believe that our in-depth exploration of the resource provided here may contribute to the discovery of other potential β-cell-specific diagnostic and therapeutic targets for diabetes.

## Figures and Tables

**Table 1:** The list of leading signaling pathways mediated by *DMT1*-KD (*p* < 0.05).

Signaling Pathway	N	Gene Names
Autophagy	22	PIK3C3; RNF185; EIF4G1; CTTN; DDIT3; STING; BAG3; DAP; IRGFM1; HMGB1; MTCL1; SQSTM1; LZTS1; ATG4C; DNM1L; HSP90AB1; GPSM1; ABL1; MAPT; NUPR1; MTDH; SUPT5
Macroautophagy	6	PIK3C3; STING; SQSTM1; LZTS1; GPSM1; SUPT5
ERAD pathway	4	RNF185; RHBDD1; BRSK1; OS9
Cell Cycle Arrest	7	PPP2R5B; DDIT3; TBRG1; HSP90AB1; BIN1; CAST; FOXM1
Wnt Signaling	28	FZD6; DDIT3; PRICKLE1; WNK2; AXIN1; UBR5; MAPK14; SLC9A3R1; CD44; CSNK1G2; CTTNBIP1; RSPO4; CUSTOS; CSNK1D; HDAC1; CTNND1; CTTND2; LEO1; ABL1; HNF1A; TAX1BP3; CTR9; EMD; RF1; CSNK1G3; MARK2; CSNK1A1; SOX9
STAT Signaling	10	SPHK2; SMPD3; CD44; FAS; STAT1; STAT3; STAT6; SOX9; HDAC1; CTR9
JNK pathway	7	MAP3K5; IL1F9; AXIN1; HMGB1; PJA2; MINK1; TAOK1;
ROS biosynthetic/metabolic process	9	NDUFA13; SPHK2; SMPD3; MAPK14; COA8; SIRT3; HDAC4; PXN; FOXM1
Apoptotic signaling pathway	37	LMNA; MAP3K5; CTTN; ICAM1; DDIT3; NDUFA13; PDCD10; VDR; SLC35F6; BAG3; DAP; TRIAP1; YBX3; HMGB2; ATP2A1; TRPS1; SLC9A3R1; CD44; COA8; ATAD5; MAPK7; FAS; BCLAF1; SCG2; ZC3HC1; DNM1L; BRSK2; RPS3; HDAC1; BIRC6; MAPT; NUPR1; MELK; PTPN1; VEGFA; CDK11B; FACTO
Inflammatory response	17	ICAM1; STING; IL1F9; HMGB1; HMGB2; MAPK14; CD44; CTNNBIP1; SMPDL3B; CDK19; PJA2; NPY; NUPR1; HCN2; RICTOR; SLC7A2; HNRNPA0
Ubiquitin-proteasome system (UPS)	32	RNF185; DDIT3; PRICKLE1; PCNP; USP37; PSMA5; AXIN1; RNF4; OTUD5; PSMC2; RNF168; AURKA; UBE2C’ UBE2S; MTA1; SUMO3; PSMA7; RYBP; CSNK1D; NSFL1C; HSP90AB1; KAT7; BIRC6; TRIP12; UBA1; UHRF1; USP48; OS9; MAP1A; USP1; RNF4; CSNK1A1
DNA repair	23	NPM1; CLSPN; UBR5; PTTG1; MCRS1; RNF168; RFC1; XRN2; TAOK1; RPS3; PAGR1; TRIP12; CHEK1; ABL1; BOD1L; UHRF1; TRIM28; UBR5; SMG1; USP1; FOXM1; SMC3; LIG3
NF-Kb signaling	7	NPM1; ICAM1; DAP; PTMA; KRAS; RPS3; MTDH
Insulin secretion	18	IRS2; PCLO; BAIAP3; SYTL4; HMGCR; CPLX1; MAFA; RIMS2; ICA1; SIRT3; BMP8A; BRSK2; VGF; HDAC1; HNF1A; PTPRN2; NOC2; MYT1
Glucose homeostasis	8	ALMS1; CACNA1A; NUCKS1; VGF; NCOR2; HNF1A; NOC2; MYT1
Cholesterol homeostasis	8	ABCA1; OSBP; HMGCR; VDR; ALMS1; HMGCS1; HNF1A; RALY
Sodium ion transport	11	SLC38A4; WNK2; SLCO1C1; GNAS; PCP4; SLC9A3R1; NEFH; COMT; ANK3; HNRNPA1; HCN2
Calcium ion transport	15	CACNB2; TRPM7; ICAM1; MCUR1; VDR; ATP2A1; CBARP; CACNA1A; LETM1; DNM1L; PANX1; CACNA1H; BIN1; STIM1; ANK2

## Data Availability

Proteomics and phosphoproteomics data are available via ProteomeXchange with the identifier PXD017216.

## Supplementary Materials

The following are available online at https://www.mdpi.com/article/10.3390/ijms22158013/s1.

## Abbreviations

T1D	type 1 diabetes
T2D	type 2 diabetes
IL-1β	interleukin-1 beta
TNF	tumor necrosis factor
IFN	interferon
NF-κB	nuclear factor kappa B
ROS	reactive oxygen species
NGF	nerve growth factor
TLR	toll-like-receptor
ERAD	endoplasmic reticulum-associated degradation
JNK	c-Jun N-terminal kinase
siRNA	small interfering RNA
ER	endoplasmic reticulum
LC-MS/MS	liquid chromatography-tandem mass spectrometry
TMT	tandem mass tags
INS-1E	insulin-producing β-cell line
FDR	false discovery rate
mRNA	messenger RNA
ADAM	disintegrin and metalloproteinase domain-containing protein
CON	un-transfected control β-cells
SC	scrambled siRNA transfected control β-cells
DMT1-KD	control β-cells transfected with three different siRNAs targeting DMT1
CON-IL	un-transfected β-cells exposed to IL-1β
SC-IL	scrambled siRNA transfected β-cells exposed to IL-1β
DMT1-KD-IL	β-cells exposed to IL-1β transfected with three different siRNAs targeting DMT1
