# Supplementary material for: Divalent Metal Transporter 1 Knock-Down Modulates IL-1β Mediated Pancreatic Beta-Cell Pro-Apoptotic Signaling Pathways through the Autophagic Machinery
Source: Int J Mol Sci. 2021 Jul 27;22(15):8013. doi: 10.3390/ijms22158013 (PMC8348373; doi:10.3390/ijms22158013)
Supplement: Supplementary file 1 [file ijms-22-08013-s001.zip › FigureS1.pdf]

Divalent Metal Transporter 1 knock-down modulates IL-1β mediated pancreatic beta-cell pro-apoptotic signaling pathways through the autophagic machinery

Taewook Kang[1,2], Honggang Huang[1], Thomas Mandrup-Poulsen[3], Martin R. Larsen[1,*]

[1] Protein research group, Department of Biochemistry and Molecular Biology, University of Southern Denmark, 5230 Odense M, Denmark.

[2] The Danish Diabetes Academy, Odense, Denmark

[3] Immuno-endocrinology Laboratory, Department of Biomedical Sciences, University of Copenhagen, 2200 Copenhagen N, Denmark.

***Lead contact: Prof. Dr. Martin R. Larsen:**

Department of Biochemistry and Molecular Biology, the University of Southern Denmark, Denmark.

Tel.: 45-60-111872; E-mail: mrl@bmb.sdu.dk

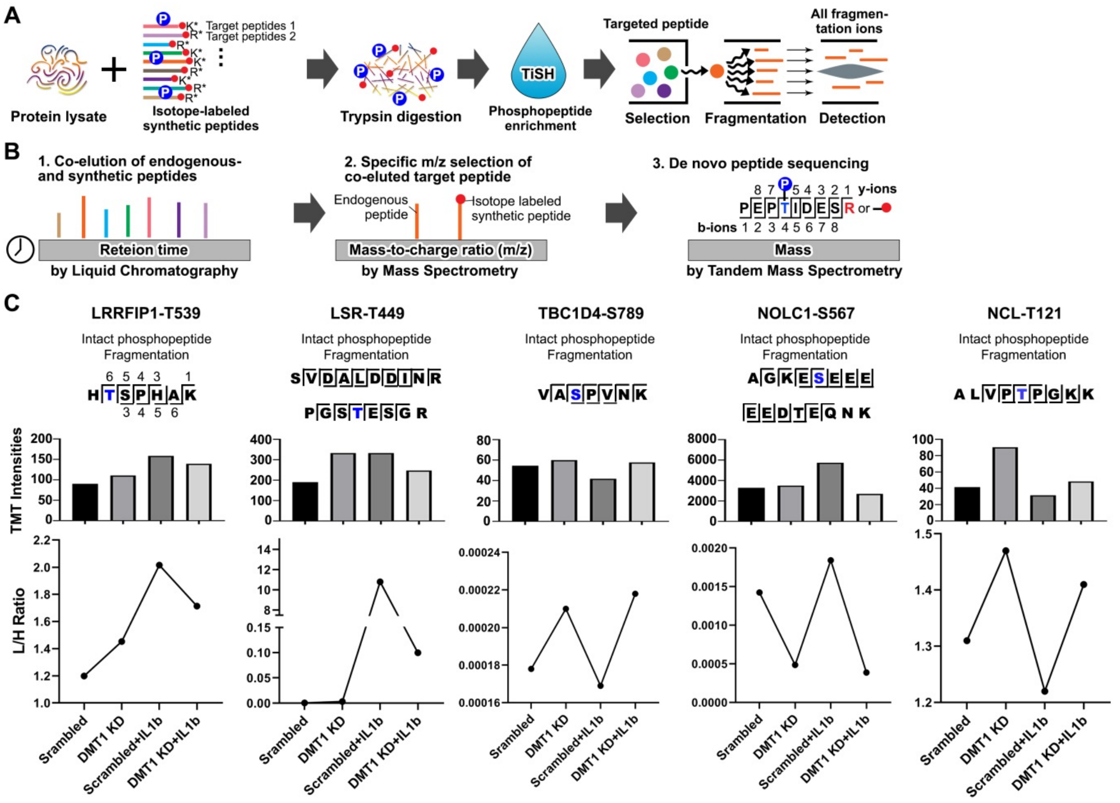

Figure S1. PRM analysis of the potential protective phosphoproteins against IL-1β by suppressing DMT1 in β-cells.
